# Plasma GDF-15 concentration is not elevated in open-angle glaucoma

**DOI:** 10.1371/journal.pone.0252630

**Published:** 2021-05-28

**Authors:** Wouter H. G. Hubens, Mariëlle T. Kievit, Tos T. J. M. Berendschot, Irenaeus F. M. de Coo, Hubert J. M. Smeets, Carroll A. B. Webers, Theo G. M. F. Gorgels

**Affiliations:** 1 University Eye Clinic Maastricht, Maastricht University Medical Center, Maastricht, The Netherlands; 2 School for Mental Health and Neuroscience, Maastricht University, Maastricht, The Netherlands; 3 Department of Biomedical Engineering, Eindhoven University of Technology, Eindhoven, The Netherlands; 4 Department of Toxicogenomics, Maastricht University, Maastricht, The Netherlands; Duke University, UNITED STATES

## Abstract

**Aim:**

Recently, the level of growth differentiation factor 15 (GDF-15) in blood, was proposed as biomarker to detect mitochondrial dysfunction. In the current study, we evaluate this biomarker in open-angle glaucoma (OAG), as there is increasing evidence that mitochondrial dysfunction plays a role in the pathophysiology of this disease.

**Methods:**

Plasma GDF-15 concentrations were measured with ELISA in 200 OAG patients and 61 age-matched controls (cataract without glaucoma). The OAG patient group consisted of high tension glaucoma (HTG; n = 162) and normal tension glaucoma (NTG; n = 38). Groups were compared using the Kruskal-Wallis nonparametric test with Dunn’s multiple comparison post-hoc correction. GDF-15 concentration was corrected for confounders identified with forward linear regression models.

**Results:**

Before correcting for confounders, median plasma GDF-15 levels was significantly lower in the combined OAG group (p = 0.04), but not when analysing HTG and NTG patients separately. Forward linear regression analysis showed that age, gender, smoking and systemic hypertension were significant confounders affecting GDF-15 levels. After correction for these confounders, GDF-15 levels in OAG patients were no longer significantly different from controls. Subgroup analysis of the glaucoma patients did not show a correlation between disease severity and plasma GDF-15, but did reveal that for NTG patients, intake of dietary supplements, which potentially improve mitochondrial function, correlated with lower plasma GDF-15.

**Conclusion:**

The present study suggests that plasma GDF-15 is not suited as biomarker of mitochondrial dysfunction in OAG patients.

## Introduction

Glaucoma is the leading cause of irreversible blindness worldwide [1]. The disease is characterized by loss of retinal ganglion cells (RGC), the cells that transfer visual information from the eye to the brain. RGC loss is often asymptomatic in early stages, but gradual progression can lead to permanent blindness [2]. 74% of all glaucoma patients have primary open-angle glaucoma (POAG), which affects over 65 million individuals.[3] Risk factors for POAG are aging, black ethnicity, male gender, genetic composition and high intraocular pressure (IOP) [2–5]. With respect to elevated IOP, there is some debate in the literature as to whether patients without elevated IOP, i.e. normal tension glaucoma (NTG), constitute as separate type of open-angle glaucoma (OAG), or whether POAG represents a disease spectrum which includes both high tension glaucoma (HTG) and NTG [6,7]. Independent of the classification of NTG as a separate OAG or as a subset of POAG, there is consensus that it is important to distinguish between the two glaucomatous optic neuropathies, as the underlying pathogenesis may differ. In this manuscript, we use the term OAG, to refer to the combined group of HTG and NTG patients.

The pathophysiology of OAG is not known in detail. There is increasing evidence from several sources that mitochondrial dysfunction plays a role in OAG. In general, it is known that mitochondrial function declines with age, and in OAG, age is both a risk factor as well as a prognostic factor [8]. More specifically, genetic studies suggest that genes encoding mitochondrial proteins contribute to OAG risk [9,10]. This includes sequence variants and mutations in genes such as *TBK1*, *OPTN*, *OPA1*, *MFN1*, *MFN2*, *TXRND2*, *PARL*. Also biochemical analysis of affected tissue points at involvement of mitochondria: post-mortem trabecular meshwork tissue of OAG patients contains more deletions in the mitochondrial genome (mtDNA); less mitochondria per cell as assessed by the mtDNA/nuclear DNA ratio [11,12]; and pathway analysis of differentially expressed proteins in the retina of post-mortem OAG and control eyes, indicate defects in oxidative phosphorylation in OAG [13].

Recently, detailed studies in a mouse model of glaucoma, the DBA/2J mouse, demonstrated that mitochondrial dysfunction in the retina is an early feature in this glaucoma model [14,15]. Strikingly, bolstering mitochondrial function by administration of nicotinamide (the amide of vitamin B3), robustly protected the mice from glaucoma. Very recent results of a clinical trial suggest that OAG patients can also benefit from this therapeutic strategy, as supplementation of nicotinamide improves their inner retinal function [16].

In view of this attractive, new therapeutic opportunity, it is now very important to establish whether mitochondrial dysfunction is involved in OAG, and if so, whether this involves all patients or only a subgroup. To investigate this, a biomarker for mitochondrial dysfunction is required that can be measured in readily accessible tissue e.g. blood, since obtaining a retinal biopsy is not possible. Recently, blood levels of growth differentiation factor-15 (GDF-15) have been described as a biomarker to diagnose mitochondrial dysfunction, with an estimated average sensitivity vs. specificity of 89.7% to identify primary mitochondrial disease patients [17–24].

In the present study, we used this biomarker. By measuring plasma GDF-15 in HTG and NTG patients, and controls, we aimed to gather indications on the role of mitochondrial dysfunction in glaucoma. In addition, we aimed to determine whether plasma GDF-15 levels would help to identify glaucoma patients that have distinct mitochondrial dysfunction.

## Methods

### Samples

EDTA plasma samples were obtained from the Eye Tissue Bank Maastricht (ETBM). The ETBM collects and stores blood from glaucoma and cataract patients of the University Eye Clinic Maastricht. The study population thus consists of patients living in the southern part of the Netherlands. All patients, were diagnosed by experienced ophthalmologists and had signed an informed consent for use of their biomaterial for scientific studies. The ethical committee of Maastricht University Medical Center approved the current study (approval number 2018–0935).

### Criteria

We obtained plasma from OAG patients diagnosed by glaucoma specialists using the following criteria: open anterior chamber angles on gonioscopy; no physical abnormalities in the anterior chamber (pigmentation or pseudoexfoliation); glaucomatous optic nerve head changes. Based on the untreated IOP, OAG patients were further divided into two groups, HTG patients (>21mmHg) and NTG patients (<21 mmHg). Patients without glaucoma, diagnosed with cataract served as our control group. Clinical data related to their general health, systemic medication, eye medication, ocular disease history and eye measurements (IOP, visual field, nerve fiber thickness) were obtained from the electronic patient data system. Exclusion criteria for the current study were presence of severe systemic diseases i.e. obesity, diabetes, cancer and COPD and a history of other ocular diseases, e.g. diabetic retinopathy, age related macular degeneration or uveitis. To obtain retrospective, lifestyle data related to health, we sent the participants a questionnaire (S1 File).

### Questionnaire

Of the questionnaire (S1 File), for this manuscript, height and weight were relevant in order to calculate BMI, and the questions related to smoking behavior and over-the-counter medication/dietary supplements were relevant for their potential correlation with mitochondrial function.

### GDF-15

Plasma GDF-15 concentration was determined using a commercially available ELISA kit (EHGDF15, Invitrogen, Thermofisher Scientific, USA) according to manufacturer’s protocol. Absorbance was measured at 450nm. Concentrations were calculated using the “Four Parameter Logistic Curve” online data analysis tool (MyAssays Ltd; http://www.myassays.com/four-parameter-logistic-curve.assay).

### Statistics

Statistical analyses were performed using IBM SPSS Statistics version 25 (SPSS Inc., USA). To determine group differences for measured parameters, two-sided t-test, chi-square distribution, Mann-Whitney U test, one-way ANOVA with Bonferroni correction or Kruskal-Wallis nonparametric test with Dunn’s multiple comparison were used as indicated. Shapiro-Wilk test was used to test for normality distribution. To study predictors of plasma GDF-15 levels, GDF-15 concentrations were normalized using natural logarithmic transformation (ln) as described previously [25]. Plasma lnGDF-15 was assigned as a dependent variable and multiple linear regression models were created. The most significant parameter was manually added and the process was repeated until none of the remaining parameters were significantly related with plasma lnGDF-15 (p<0.05). GraphPad Prism 6 (GraphPad Software, USA) was used to plot the data. For correlation between blood measurements and eye measurements, we decided to calculate an average per patient using both the value of the left (OS) and right (OD) eye i.e. average ODS. We additionally calculated correlations using per patient only the measurements of the eye that was most affected i.e. eye with highest IOP, lowest visual field, thinnest average retinal nerve fiber layer thickness.

To calculate the required sample size for the detection of significant differences between OAG patients and controls, we based the expected differences on blood GDF-15 values reported previously [26]. A power calculation was performed based on these previously reported median values, with the alpha set to 0.05 and power 0.9 [27]. For this calculation, the interquartile range was used as an estimation of the standard deviation.

## Results

### Demographics

The ETBM had blood samples of 261 participants available that met our criteria as described in the methods. These samples were collected between April 2017 and November 2018 and analyzed January 2020. Detailed patient and lifestyle characteristics of these 261 patients are described in Tables 1 and 2. All were Caucasian. There was no difference in average age and gender between the groups.

**Table 1:** Characteristics of OAG patients and controls.

	Control	OAG	p. value (test)
Number of patients (n)	61	200	
Age (years)	70.5 ± 8.1	69.5 ± 9.4	0.451 (t-test)
Gender (male/female)	31/30	110/90	0.566 (chi-square)
BMI	26.2 ± 4.0	24.9 ± 3.2	**0.007** (t-test)
Hypertension (%)	26 (43%)	53 (27%)	**0.016** (chi-square)
Hypercholesterolemia (%)	10 (16%)	17 (9%)	0.076 (chi-square)
Smoking	5 (8%)	10 (5%)	0.348 (chi-square)
Neurological disorder	6 (10%)	9 (5%)	0.117 (chi-square)
Vascular disease	9 (15%)	21 (11%)	0.362 (chi-square)
Dietary supplements	7 (11%)	42 (21%)	0.105 (chi-square)
IOP ODS (mmHg)	16.4 ± 3.9	13.5 ± 3.9	**<0.001** (t-test)
IOP highest eye (mmHg)	17.9 ± 4.9	14.7 ± 4.6	**<0.001** (t-test)
mD ODS (dB)	NA	-10.0 ± 7.2	NA
mD most progressed eye (dB)	NA	-13.4 ± 8.3	NA
RNFLT ODS (μm)	NA	61.9 ± 13.2	NA
RNFTL most progressed eye (μm)	NA	53.6 ± 15.6	NA

Values are expressed as mean ± standard deviation. Ocular values are either an average calculated from the value of both Oculus Dextra (OD) and Oculus Sinistra (OS) (ODS) or the value of the eye that is most affected. Bold highlights significant p-values. BMI: Body mass index; IOP: Intraocular pressure (treated); mD: Mean deviation of visual field; RNFLT: Retinal nerve fiber layer thickness; BMI: Body mass index.

**Table 2:** Characteristics of the patient groups with OAG patients classified into HTG and NTG.

	Control	HTG	NTG	p. value (test)
Number of patients (n)	61	162	38	
Age (years)	70.5 ± 8.1	69.4 ± 9.8	70.1 ± 7.4	0.695 (ANOVA)
Gender (male/female)	31/30	93/69	17/21	0.314 (chi-square)
BMI	26.2 ± 4.0	24.9 ± 5.4	24.7 ± 3.0	**0.017** (ANOVA)
Hypertension (%)	26 (43%)	41 (25%)	12 (32%)	**0.042** (chi-square)
Hypercholesterolemia (%)	10 (16%)	13 (8%)	4 (11%)	0.188 (chi-square)
Smoking	5 (8%)	10 (6%)	0 (0%)	0.218 (chi-square)
Neurological disorder	6 (10%)	9 (6%)	0 (0%)	0.122 (chi-square)
Vascular disease	9 (15%)	18 (11%)	3 (8%)	0.564 (chi-square)
Dietary supplements	7 (11%)	32 (20%)	10 (26%)	0.184 (chi-square)
IOP ODS (mmHg)	16.4 ± 3.9	14.0 ± 4.0	11.3 ± 2.8	**<0.001** (ANOVA)
IOP highest eye (mmHg)	17.9 ± 4.9	15.2 ± 4.6	12.6 ± 3.6	**<0.001** (ANOVA)
mD ODS (dB)	NA	-11.0 ± 7.9	-11.6 ± 7.0	0.613(t-test)
mD most progressed eye (dB)	NA	-14.3 ± 8.8	-14.7 ± 7.8	0.333 (t-test)
RNFLT ODS (μm)	NA	62.3 ± 12.8	60.0 ± 14.9	0.492 (t-test)
RNFTL most progressed eye (μm)	NA	52.7 ± 16.2	53.4 ± 18.8	0.225 (t-test)

Values are expressed as mean ± standard deviation. Ocular values are either an average calculated from the value of both Oculus Dextra (OD) and Oculus Sinistra (OS) (ODS) or the value of the eye that is most affected. Bold highlights significant p-values. HTG: High tension glaucoma; NTG: Normal tension glaucoma; BMI: Body mass index; IOP: Intraocular pressure (treated); mD: Mean deviation of visual field; RNFLT: Retinal nerve fiber layer thickness; BMI: Body mass index.

### Ocular characteristics

Glaucoma patients were taking medication to lower IOP. Treated IOP of OAG was significantly lower than IOP of controls (Table 1; p<0.001). This was due to the inclusion of NTG patients, as IOP of HTG patients was not significantly different from controls (Table 2; p<0.001). Average disease severities of HTG and NTG patients were similar (Table 2).

### Systemic factors

The control group had significantly higher BMI and prevalence of systemic hypertension (p = 0.007 and p = 0.017). The prevalence of patients with either vascular (e.g. pacemaker, coronary bypass history) or neurological disorders (e.g. depression, dementia) was relatively low and did not differ between groups.

### Lifestyle

Ten patients (4 controls, 6 HTG) were current smokers with an average of 10 cigarettes a day. As it may take time to recover from the effects of long time smoking we also included five patients that recently quit smoking i.e. quit within the last year. On average, these patients used to smoke 12 cigarettes per day. There was no difference in prevalence of smokers and recent smokers between groups. Considering the small sample size, we combined the two smoking groups. In addition, we asked the patients to specify their use of over-the-counter dietary supplements. Since our goal was to measure GDF-15 in relation to mitochondrial dysfunction, in our analysis we only considered supplements that, based on known literature, may improve mitochondrial function. A list of these supplements is provided in S1 Table. As most of these supplements contain more than 100% of the daily-recommended dose of the nutrients, we scored supplements on a yes/no basis. This meant that in our analysis we did not differentiate between patients that took multiple supplements (e.g. multivitamin tablets and vitamin b complex) and patients that took a single supplement. The number of patients that took at least one dietary supplement did not differ between the groups.

#### Plasma GDF-15

**Fig 1:**
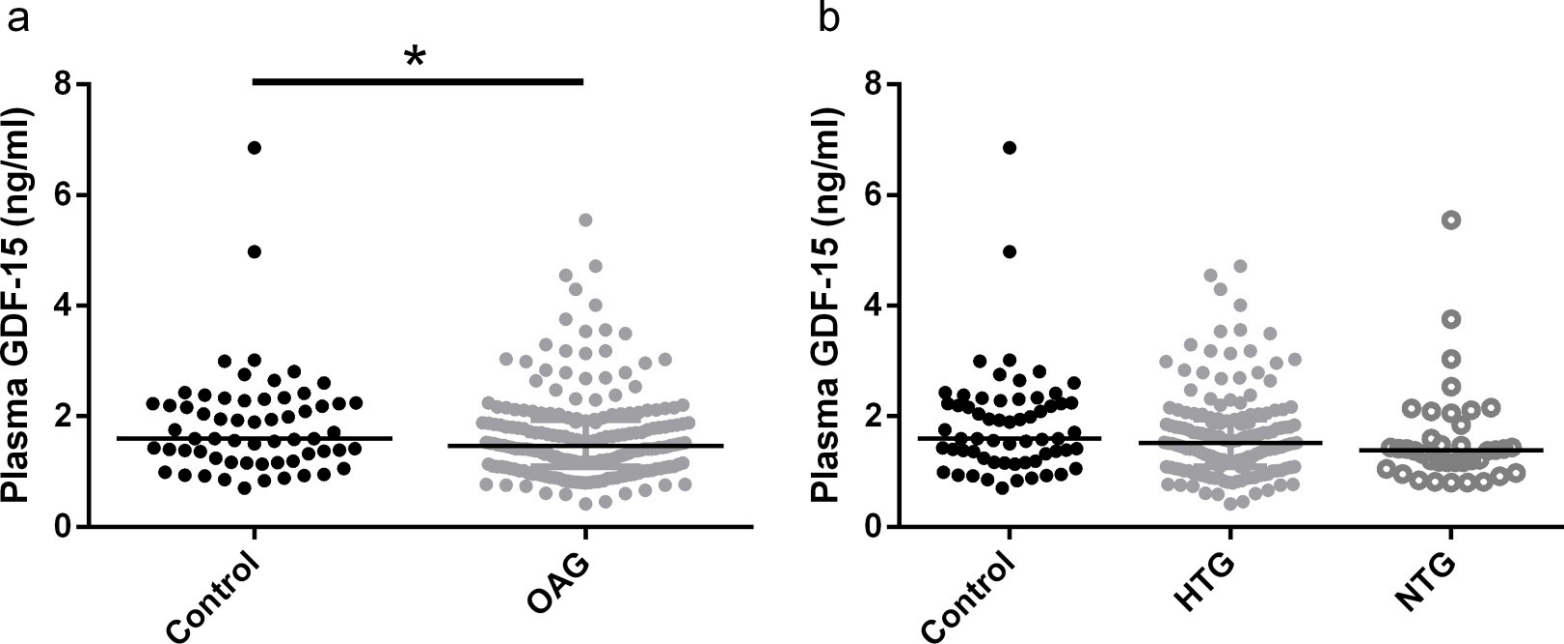
Median plasma GDF-15 concentration of controls vs. OAG (a) and of controls vs HTG and NTG patients (b). Median (interquartile range) of control: 1.60 ng/ml (1.22–2.26); OAG: 1.46 ng/ml (1.09–1.94); HTG: 1.52 ng/ml (1.09–1.95) and NTG: 1.38 ng/ml (1.14–1.90). OAG: Open-angle glaucoma; HTG: High tension glaucoma; NTG: Normal tension glaucoma.

According to the power calculation, 21 patients per group should be sufficient to detect significant differences in plasma GDF-15 concentrations, which is well below our group sizes of 61 for the control and 200 for the OAG group, composed of 162 HTG and 38 NTG patients. In our initial analysis, we compared median GDF-15 levels by Mann-Whitney U test, as the plasma GDF-15 concentration was not normally distributed (p<0.0001). Median plasma GDF-15 was significantly lower in OAG patients compared to controls (Fig 1A; p = 0.04). With respect to the OAG group, no significant differences were observed between HTG and NTG patients (Fig 1B; p = 0.99). In addition, neither HTG (p = 0.22) nor NTG patients (p = 0.09) differed significantly from controls in this separate analysis (Fig 1B).

**Fig 2:**
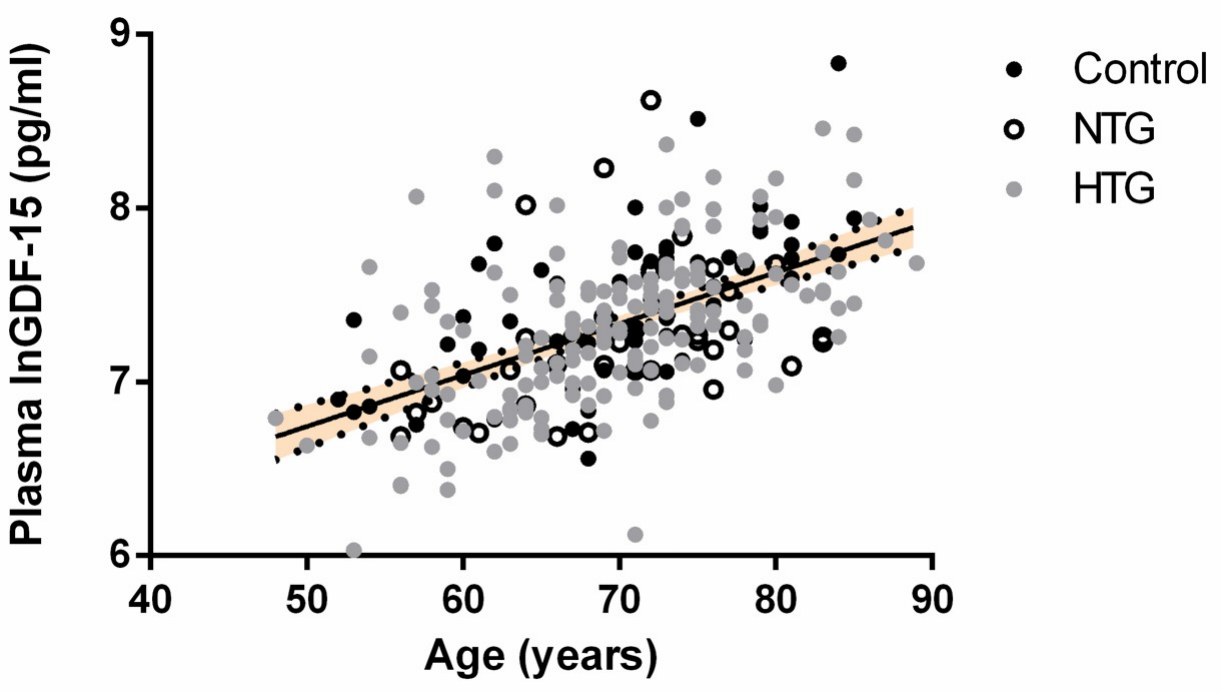
Correlation between plasma GDF-15 concentration (Ln transformed pg/ml) and age in a univariate linear regression model. LnGDF-15 was significantly correlated with age (β = 0.532; p<0.001).

**Fig 3:**
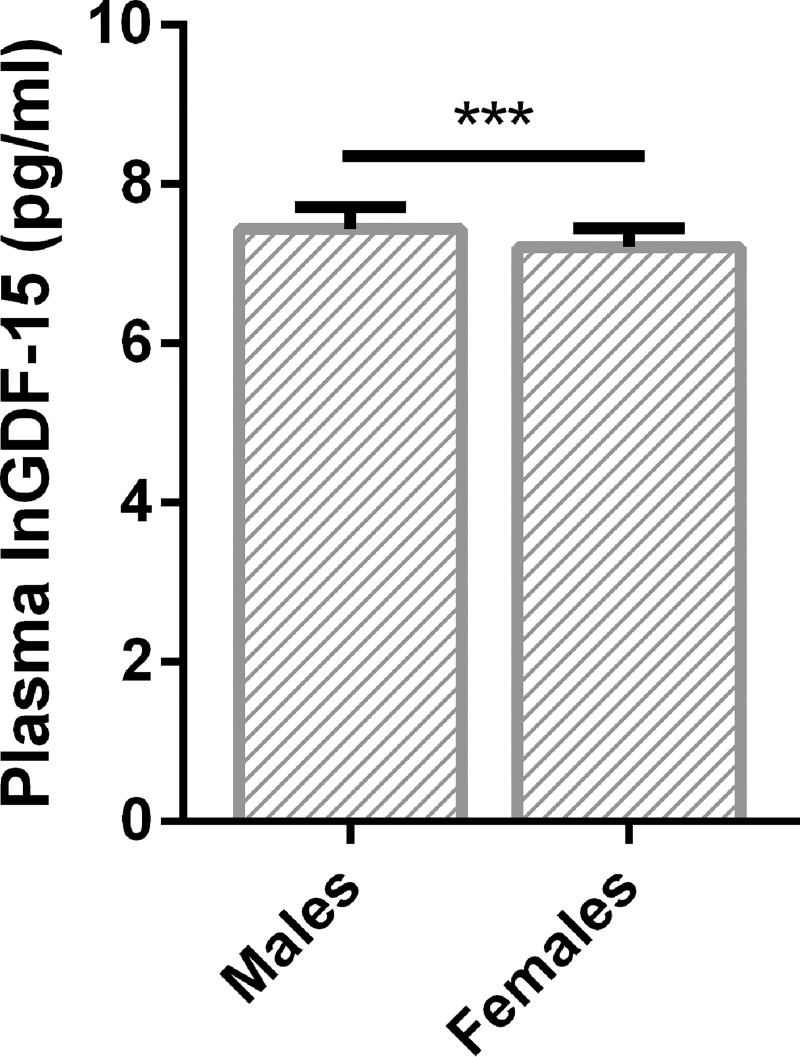
Gender differences in plasma GDF-15 concentration (Ln transformed pg/ml). Males had significantly higher LnGDF-15 compared to females. Median and interquartile range are depicted. *** p<0.001.

**Fig 4:**
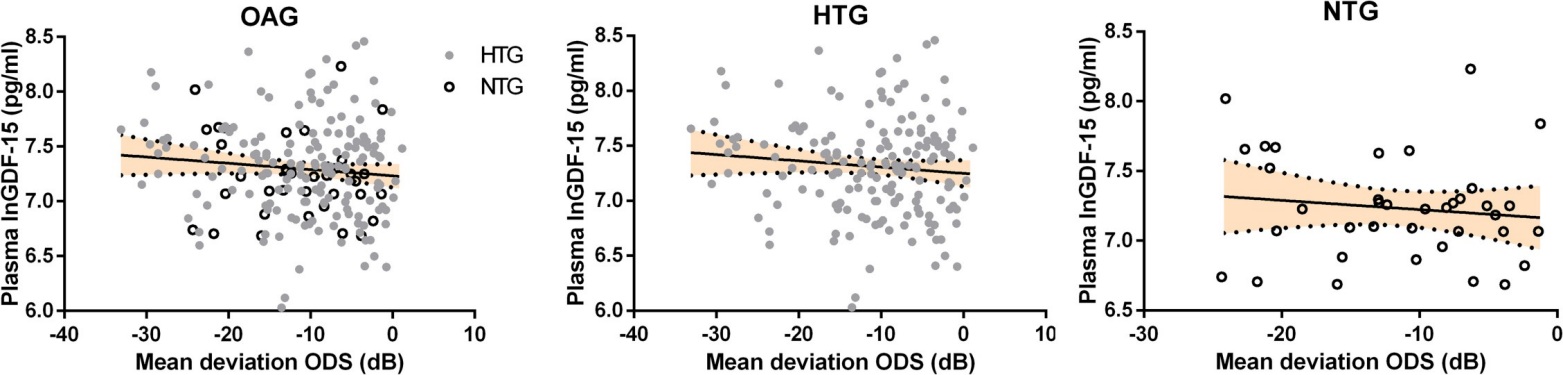
Correlation between plasma GDF-15 concentration (Ln transformed pg/ml) and OAG disease severity. Plasma LnGDF-15 did not correlate with OAG disease severity as assessed by the mean deviation of the average of both eyes (ODS). Neither in the combined OAG population (β = -0.100; p = 0.159), nor when HTG (β = -0.099; p = 0.208) and NTG (β = -0.122; p = 0.470) patients were assessed separately.

Next, we determined which demographic or lifestyle factors had an influence on the concentration of plasma GDF-15. This was done using a univariate linear regression analysis with natural logarithmic transformed GDF-15 (lnGDF-15) as dependent variable. In line with our initial analysis, OAG diagnosis was negatively associated with lnGDF-15 concentration (p<0.05). Much stronger correlations were observed between lnGDF-15 concentration and age (Fig 2; p<0.001), between lnGDF-15 and gender (Fig 3; p<0.001) and between lnGDF-15 and presence of systemic hypertension (p<0.001). Lastly, BMI (p<0.05) was also correlated with lnGDF-15. No significant relation between lnGDF-15 and disease severity was observed (Fig 4).

To correct our initial results for these possible confounders, we incorporated them in a multiple linear regression model in a forward conditional manner (Table 3). The final model with age, gender, smoking and systemic hypertension as covariates explains 30.6% of the variance in plasma lnGDF-15 concentration. Of note, in this multiple regression model, OAG diagnosis no longer has a significant contribution in explaining the variance in plasma lnGDF-15 concentration.

**Table 3:** Multiple linear regression model with lnGDF-15 as dependent variable.

Model		Unstandardized β	Std. Error	Standardized β	t	p-value
1	(Constant)	5.838	0.192		30.430	<0.001
	Age	0.022	0.003	0.450	8.130	<0.001
	Gender	-0.208	0.046	-0.233	-4.473	<0.001
	Smoking	0.294	0.102	0.154	2.893	0.004
	HT	0.121	0.052	0.125	2.336	0.020

For gender females were coded as 1. HT; systemic hypertension. Model Adjusted R^2^ = 0.306.

In addition, we performed multiple linear regression models for the glaucoma subgroups to determine if there were any differences between HTG and NTG that may have been obscured in the combined model. Similar to the combined model, lnGDF-15 concentration in HTG patients was significantly correlated with age, gender and systemic hypertension (S2 Table). Smoking was excluded from the HTG model, being almost significant (p = 0.069). Interestingly, in NTG patients we observed a significant negative correlation between lnGDF-15 and self-reported intake of dietary supplements, that potentially bolster mitochondria (S3 Table; p<0.05).

## Discussion

We performed this study on plasma levels of GDF-15, a relatively new biomarker for mitochondrial dysfunction, to gather indications that mitochondrial dysfunction plays a role in glaucoma and to determine whether plasma GDF-15 levels would help to identify a subset of glaucoma patients that have mitochondrial dysfunction. Measurements were done in 38 NTG patients, 162 HTG patients and 61 age-matched controls. Corrected for identified confounders, no differences were found between these groups, nor did we identify glaucoma patients with a distinct, mitochondrial dysfunction.

### Study strength and limitations

Our study population was quite large, included NTG as well as HTG patients, and used linear regression models for detailed analysis of the relation between plasma GDF-15 concentration and glaucoma. We additionally included a questionnaire to get more insight in potential confounding lifestyle factors. As GDF-15 measurements can be affected by systemic diseases such as diabetes and COPD [28–30], patients with these diseases were excluded. Other potential confounders are more difficult to exclude, for example, prescribed ocular glaucoma medication. Although systemic side-effects for ocular medication are uncommon, they can occur [31]. Currently, it is not known whether glaucoma eye drops have an effect on blood GDF-15 levels. Another possible limitation of our study was that blood of patients was collected during routine visits to the clinic. Therefore, the fasting conditions, i.e. time between last food intake and blood sampling, can vary substantially. Fortunately, GDF-15 plasma levels are relatively stable throughout the day with no substantial postprandial increase in concentration [32,33]. In addition, we have to take into account that the ocular measurements to assess glaucoma, e.g. visual field measurement, were not always performed at the same day as the blood collection. As an extra test to check whether these correlations with plasma GDF-15 were reliable, we additionally performed regression analyses of 34 OAG patients of which blood collection and ocular measurements were performed at the same day. In line with the results of our entire cohort, there was no significant correlation between glaucoma disease severity and plasma lnGDF-15 levels in these patients.

### Factors that influence plasma GDF-15 concentration

Our multiple linear regression model revealed that age, gender, smoking and systemic hypertension significantly influenced plasma GDF-15. A correlation between age and GDF-15 has been reported previously [25,34–36]. Independent of the disease, males had significantly higher plasma GDF-15 than females (Figs 3 and S1). Studies on other diseases also report higher GDF-15 expression in males [37]. There might be an innate difference in regulation of stress response expression of GDF-15 in the different genders [38]. As we observed significant gender differences, we additionally analyzed males and females separately (S1 Fig). This analysis showed that the median plasma GDF-15 concentration did not significantly differ between controls, HTG and NTG, in males (2090pg/ml vs. 1645 pg/ml vs. 1483 pg/ml) nor in females (1407pg/ml vs. 1419 pg/ml vs. 1172 pg/ml). A correlation between GDF-15 and blood pressure c.q. hypertension also has been reported previously [39]. As expected, smoking was significantly positively correlated with plasma GDF-15. Inhaled cigarette smoke is known to induce mitochondrial respiratory chain dysfunction in blood cells and can induce GDF-15 expression in lung cells [40,41].

We additionally aimed to control for some supplements that, based on literature, could bolster mitochondrial function (S1 Table). Noted, it is difficult to correct for this factor precisely, for example, given that these data were self-reported and difficult to quantify. Nonetheless, after combining all of these different dietary supplements into a mitochondrial category on a yes/no basis, we found that the use of these supplements negatively correlated with plasma lnGDF-15 concentration in NTG patients (S3 Table; p<0.05). While acknowledging all uncertainties associated with this self-reported factor, this finding is in line with the hypothesis that mitochondrial dysfunction is implicated in OAG, especially in NTG patients. Dietary supplementation as therapeutic strategy merits further investigation, as it would be easy to implement in clinical practice.

### Previous findings

To our knowledge, with respect to glaucoma, there has been one previous GDF-15 study on aqueous humor (AH) and one on serum [26,42]. In AH, a significant and very strong increase in median GDF-15 concentration was observed compared to controls (63.4 pg/ml in POAG vs. 2.0 pg/ml in control) [42]. Also in the study on the serum, a significant, albeit much smaller, increase in median GDF-15 concentration was observed compared to controls, (1.04 ng/ml in HTG vs. 0.74 ng/ml in controls) [26]. In contrast to this study by Bourouki et al., our statistical analysis did not reveal significant differences in plasma GDF-15. Obviously, there are differences between the two studies that may influence the outcome, for example, differences in blood sample type (serum vs. EDTA plasma) and ELISA kit (not specified vs. Invitrogen). With regard to the study groups (Greek vs. Dutch), there was a difference in sample size (57 HTG and 44 controls vs. 162 HTG, 61 controls), while inclusion and exclusion criteria were similar, though not identical. In this respect, it may be relevant that Bourouki et al. did not correct for confounders in their statistical analysis. In our study group, this made a clear difference: we identified age (Fig 2) and gender (Fig 3) as significant confounders, despite age and gender not differing significantly between the groups. In addition, presence of hypertension and lifestyle factors i.e. smoking and self-reported intake of dietary supplements (by NTG patients in a subgroup analysis) contributed significantly to the variance in plasma GDF-15 concentration. After correction for these factors, our initial finding of a statistically significant difference in GDF-15 levels had disappeared. While patients with hypertension and smokers were excluded in the study of Bourouki et al, other important confounders might have been identified. For instance, in our study group, when adhering to the same criteria to exclude hypertension and smokers, age and gender remained significant confounders. In addition, in this subgroup, BMI significantly influenced blood GDF-15 levels (p = 0.007). Differences in these parameters across the groups may well have influenced the outcome of the previous study.

### Plasma GDF-15 is not elevated in OAG

After carefully evaluating and correcting for confounders of plasma GDF-15 levels, we did not find any differences in between OAG patients and controls. While median plasma GDF-15 levels did not differ, it remained possible that GDF-15 levels could identify a subgroup of patients with distinct mitochondrial dysfunction. The distribution of the GDF-15 levels as shown in Fig 1, did not suggest that such a subgroup with very high GDF-15 levels exists. Yet, to more formally test this, we assessed the presence of such outliers in two ways: 1) We define outliers as those values that are more than 1.5 times the interquartile range below the first quartile or more than 1.5 times the interquartile range above the third quartile; and 2) we define outliers as values that are higher than the value of the 95^th^ percentile of the control group. Chi-square tests revealed that the 11 outliers based on interquartile ranges (p = 0.887), and 18 outliers based on the 95^th^ percentile of the control group (p = 0.780) were distributed evenly across the three groups. This suggests that plasma GDF-15 is not useful to reveal a, possibly existing, subgroup of OAG patients with a critical mitochondrial dysfunction.

There are several ways to interpret our main finding that we did not find a difference in plasma GDF-15 concentration between our glaucoma groups and controls. One explanation could be that mitochondrial dysfunction does not play a role in the pathology of glaucoma. This reasoning is very unlikely, given the accumulated evidence on the role of mitochondria in glaucoma [9,11–16,43–48]. Alternatively, an explanation could be that GDF-15 is not a suitable biomarker. Plasma GDF-15 is currently one of the most sensitive biomarkers to detect mitochondrial dysfunction, with an average sensitivity vs specificity of 89.7% to identify mitochondrial disease patients [17–24]. However, most of the mitochondrial disease patients with abnormal GDF-15 levels had a myopathy i.e. mitochondrial dysfunction in muscle cells [24]. It is known that GDF-15 in the blood originates from visceral organs such as the liver and that these organs can strongly increase expression of GDF-15 in response to signaling molecules from muscle cells affected by mitochondrial dysfunction as a result of mtDNA translational defects or mtDNA deletions [49,50]. For non-muscle affected mitochondrial diseases, the relation with plasma GDF-15 levels is less clear. In this context, Leber Hereditary Optic Neuropathy (LHON) patients form an interesting group [51,52]. Similar to glaucoma, LHON patients suffer from specific loss of RGCs, which in these patients, is the result of mitochondrial dysfunction caused by mutations in mtDNA. A recent study quantified plasma GDF-15 in a wide variety of mitochondrial diseases, including 23 adult LHON patients [23]. The study reported a plasma GDF-15 concentration range between 665 and 3300 pg/ml in LHON patients, which was not significantly different from controls.

This indicates that plasma GDF-15 clearly has limitations as a biomarker for mitochondrial dysfunction in general, and certainly does not always accurately reflect mitochondrial dysfunction of ocular tissues. It is possible that if only ocular tissue is affected, blood levels of GDF-15 may not rise detectably, especially since there are so many confounders that influence blood GDF-15 levels. Maybe then, ocular measurements of GDF-15 are more informative. Indeed, an approximately 60-fold increase in GDF-15 was observed in glaucomatous AH, suggesting that measuring GDF-15 to demonstrate a role for mitochondrial dysfunction in a glaucoma patient may still be valid option, but has to be done in local tissue [42].

To conclude, the present study suggests that plasma GDF-15 is not suited as biomarker to indicate OAG patients with mitochondrial dysfunction.

## Supporting information

S1 Fig Gender subgroup analysis. GDF-15 concentration was compared between males and females in each patient group (a) and between the three patient groups when the groups were divided by gender (b). a) For both healthy controls and NTG patients males (M) had significantly higher plasma GDF-15 concentration compared to females (F)(** p<0.01). In HTG patients a similar trend was observed (# p = 0.09). b) No differences were observed in plasma GDF-15 concentration between controls, HTG and NTG when males and females were assessed separately. Median +- interquartile range are provided. HTG: High tension glaucoma; NTG: Normal tension glaucoma.
(TIF)

S1 Table List of encountered dietary supplements that potentially bolster mitochondrial function. (DOCX)

S2 Table Multiple linear regression model for HTG patients with lnGDF-15 as dependent variable. While almost significant (p = 0.069) smoking was excluded from the model.
(DOCX)

S3 Table Multiple linear regression model for NTG patients with lnGDF-15 as dependent variable. There were no smokers among NTG patients. Self-reported intake of dietary supplements that potentially bolster mitochondrial function was the second most significant (p<0.05) confounder for plasma lnGDF-15 concentration.
(DOCX)

S1 File Questionnaire given to ETBM participants. Original language was Dutch and has been translated to English.
(PDF)

S2 File (DOCX)
