# Supplementary figures and images for: Plasma GDF-15 concentration is not elevated in open-angle glaucoma
Source: PLoS One. 2021 May 28;16(5):e0252630. doi: 10.1371/journal.pone.0252630 (PMC8162581; doi:10.1371/journal.pone.0252630)

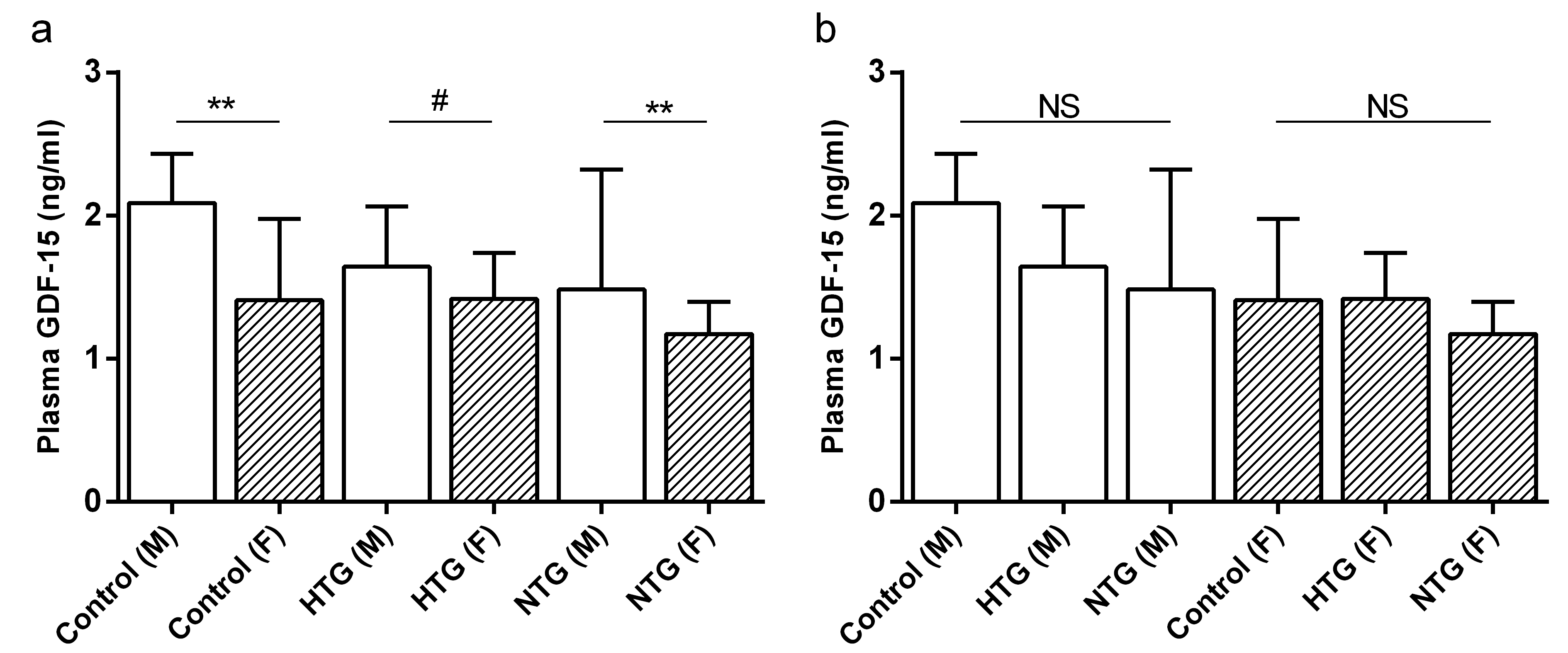

Supplement: S1 Fig — GDF-15 concentration was compared between males and females in each patient group (a) and between the three patient groups when the groups were divided by gender (b). a) For both healthy controls and NTG patients males (M) had significantly higher plasma GDF-15 concentration compared to females (F)(** p<0.01). In HTG patients a similar trend was observed (# p = 0.09). b) No differences were observed in plasma GDF-15 concentration between controls, HTG and NTG when males and females were assessed separately. Median +- interquartile range are provided. HTG: High tension glaucoma; NTG: Normal tension glaucoma. (TIF) [file pone.0252630.s001.tif]
