# Supplementary material for: Plasma GDF-15 concentration is not elevated in open-angle glaucoma
Source: PLoS One. 2021 May 28;16(5):e0252630. doi: 10.1371/journal.pone.0252630 (PMC8162581; doi:10.1371/journal.pone.0252630)
Supplement: S1 Table — (DOCX) [file pone.0252630.s002.docx]

**S1 Table.** **List of encountered dietary supplements that potentially bolster mitochondrial function.**

| Dietary supplement | Ingredient | Relation to mitochondria | Reference |
| --- | --- | --- | --- |
| Vitamin C tablets | Vitamin C | Effective scavenger of free radicals, preventing mitochondrial oxidant formation and perhaps mitochondrial aging | see review [1] |
| Hexaniacin enzymatic | Vitamin B3 analogue | Many vitamins can boost mitochondrial function | see review [1] |
| Vitamin B-complex | Vitamin B1, B2, B3, B5, B6, B8, B11 and B12 |  |  |
| Multivitamin tablet  Metarelax  Dagravit Total 30  Supradyn  Actifit Vitamin 65+  Davitamon Actifit 50+ | Variety of vitamins |  |  |
| Ginkgo Biloba  Actifit Vitamin 65+  Davitamon Actifit 50+ | Ginkgo Biloba Extract | Reduces oxidative stress and improves mitochondrial respiration | [2] |
| Supradyn | Coenzyme Q10 | Q10 is vital co-factor for oxidative phosphorylation. Supplementation can improve age-related decline in mitochondrial functioning | [[3](#_ENREF_55)] |
| Mangaan Bisglycinaat | Manganese | Required component of manganese superoxide dismutase, an important scavenger of mitochondrial reactive oxygen species | [[4](#_ENREF_56),5] |
