# Supplementary material for: Plasma GDF-15 concentration is not elevated in open-angle glaucoma
Source: PLoS One. 2021 May 28;16(5):e0252630. doi: 10.1371/journal.pone.0252630 (PMC8162581; doi:10.1371/journal.pone.0252630)
Supplement: S2 Table — While almost significant (p = 0.069) smoking was excluded from the model. (DOCX) [file pone.0252630.s003.docx]

**S2 Table.** **Multiple linear regression model for HTG patients with lnGDF-15 as dependent variable.** While almost significant (p=0.069) smoking was excluded from the model*.*

| Model |  | Unstandardized β | Std. Error | Standardized β | t | p-value |
| --- | --- | --- | --- | --- | --- | --- |
| 1 | (Constant) | 6.060 | 0.232 |  | 26.169 | <0.001 |
|  | Age | 0.018 | 0.003 | 0.398 | 5.540 | <0.001 |
|  | Gender | -0.130 | 0.063 | -0.143 | -2.053 | 0.042 |
|  | HT | 0.152 | 0.074 | 0.147 | 2.055 | 0.042 |

HT: systemic hypertension. For gender, females were coded as 1. Model Adjusted R^2^ = 0.229
