# Supplementary material for: Plasma GDF-15 concentration is not elevated in open-angle glaucoma
Source: PLoS One. 2021 May 28;16(5):e0252630. doi: 10.1371/journal.pone.0252630 (PMC8162581; doi:10.1371/journal.pone.0252630)
Supplement: S3 Table — There were no smokers among NTG patients. Self-reported intake of dietary supplements that potentially bolster mitochondrial function was the second most significant (p<0.05) confounder for plasma lnGDF-15 concentration. (DOCX) [file pone.0252630.s004.docx]

**S3 Table. Multiple linear regression model for NTG patients with lnGDF-15 as dependent variable**. There were no smokers among NTG patients. Self-reported intake of dietary supplements that potentially bolster mitochondrial function was the second most significant (p<0.05) confounder for plasma lnGDF-15 concentration.

| Model |  | Unstandardized β | Std. Error | Standardized β | t | p-value |
| --- | --- | --- | --- | --- | --- | --- |
| 1 | (Constant) | 6.458 | 0.550 |  | 26.169 | <0.001 |
|  | Gender | -0.439 | 0.110 | -0.511 | -2.053 | <0.001 |
|  | Supplements | -0.275 | 0.23 | -0.283 | -2.230 | 0.032 |
|  | Age | 0.016 | 0.008 | 0.275 | 5.540 | 0.040 |

*For gender, females were coded as 1. Model Adjusted R^2^ = 0.418*
