# Supplementary material for: Plasma GDF-15 concentration is not elevated in open-angle glaucoma
Source: PLoS One. 2021 May 28;16(5):e0252630. doi: 10.1371/journal.pone.0252630 (PMC8162581; doi:10.1371/journal.pone.0252630)
Supplement: S1 File — Original language was Dutch and has been translated to English. (PDF) [file pone.0252630.s005.pdf]

Questionnaire Eye Tissue Bank Maastricht

The answers are strictly confidential

Personal information

Length: ____cm
Weight: ____kg

Ethnicity and heritage

1. What country were your grandparents born?
   - ○ Grandma (mothers side): ______________________
   - ○ Grandpa (mothers side): ______________________
   - ○ Grandma (fathers side): ______________________
   - ○ Grandpa (fathers side): ______________________

Education

2. What is the highest level of education you completed?
   - ○ Elementary school
   - ○ Lower general secondary education (practical)
   - ○ Lower general secondary education (theoretical)
   - ○ Higher general secondary education
   - ○ Bachelor
   - ○ Master
   - ○ Doctorate/PhD

Smoking

3. Do you smoke or have smoked in the past?
   - ○ No
   - ○ Yes, I smoke since I was ___ years old
   - ○ I quit. I have smoked from my___ e till ___ e year

   When I smoke(d), I smoke(d)
   - ○ cigarettes, about ___ per day
   - ○ cigars, about ___ per day
   - ○ pipe, about ___ times per day

Alcohol

4. Do you drink alcohol?
   - ○ No
   - ○ Yes, about ___ glasses per week
   - ○ I quit. I drank from my ___ e year till my ___ e year.
     I drank about ___ glasses per day.

Dietary supplementation

5. Do you take dietary supplementation/vitamins?
   (For instance, dissolvable tablets of magnesium, calcium or vitamins)
   - ○ No
   - ○ Yes: ________________ (brand) ____ times a week
     ________________ (brand) ____ times a week
     ________________ (brand) ____ times a week
     ________________ (brand) ____ times a week

Eating behaviour

6. How often do you eat dark green vegetables (e.g. spinach, cabbage)?
   (This includes in-between snacks)
   - ○ Every day
   - ○ 4 – 6 times a week
   - ○ 3 or less times a week

   The portion size is about:
   - ○ 1 big spoon
   - ○ 2 big spoons
   - ○ 3 big spoons
   - ○ 4 big spoons

7. How often do you eat red/yellow vegetables (e.g. paprika, carrot)?
   (This includes in-between snacks)
   - ○ Every day
   - ○ 4 – 6 times a week
   - ○ 3 or less times a week

   The portion size is about:
   - ○ 1 big spoon
   - ○ 2 big spoons
   - ○ 3 big spoons
   - ○ 4 big spoons

8. How much fruit do you eat per week?
   (fruit in juice does not count)
   ○ Every day several portions
   ○ Every day 1 portion
   ○ 4 – 6 portions a week
   ○ 3 or less portions a week

9. How often do you eat fish?
   ○ Several days a week
   ○ Once a week
   ○ Sometimes (1 – 3 times a month)
   ○ (Almost) never

10. Are you on a specific diet?
   ○ No
   ○ Vegetarian
   ○ Vegan
   ○ Other, namely: ________________________

Physical activity

11. How is your physical condition and activity?
   ○ I can barely move on my own
   ○ I can move without problems, but do not move a lot.
   ○ I can walk and ride bicycle in a regular pace
   ○ I can walk and ride bicycle in an intensive pace
   ○ I regularly exercise intensively

Hereditary diseases

12. You may have visited your eye doctor for glaucoma treatment. Do you have (other) family members that are diagnosed with glaucoma?
   (if yes, fill in relation, e.g. father, sister, cousin)
   ○ No
   ○ Yes, namely: ________________________________

   ____

   ____

13. Do you have family members that are being treated for other eye diseases?
(If yes, fill in relation, e.g. father, sister, cousin)
   ○ No
   ○ Yes, namely: (family member) ________ (disease) ________

   ________ ________
   ________ ________
   ________ ________
   ________ ________
   ________ ________

Other systemic diseases

14. Are you or have you been treated for:
   ○ Diabetes (Sugar disease)
   ○ Cancer
   ○ Lung diseases
   ○ Heart attack / cardiovascular problems
   ○ Stroke
   ○ High blood pressure
   ○ Rheumatoid arthritis
   ○ Others, namely: ________

Medication

15. Do you use medication from a pharmacy that does not require a prescription (for instance, homeopathic drugs, paracetamol, aspirin)?
   ○ No
   ○ Yes, namely: ________ (brand) ____ times a week
   ________ (brand) ____ times a week
   ________ (brand) ____ times a week
   ________ (brand) ____ times a week

16. Do you give us permission to request your list of prescribed medication from your pharmacist?
   ○ No
   ○ Yes

Maastricht UMC+

Thank you for filling in this questionnaire. If you have further questions or remarks or did not have enough space to fill in specific questions, you can mention that below:

Vragenlijst Oogweefsel Bank Maastricht

De ingevulde gegevens worden strikt vertrouwelijk behandeld

Persoonsgegevens

Lengte: ____cm
Gewicht: ____kg

Etniciteit en afkomst

1. In welk land zijn uw opa/oma geboren?
   - ○ Oma (moederskant): ______________________
   - ○ Opa (moederskant): ______________________
   - ○ Oma (vaderskant): ______________________
   - ○ Opa (vaderskant): ______________________

Opleiding

2. Wat is uw hoogst afgeronde opleiding?
   - ○ Basisonderwijs
   - ○ LBO / VBO / VMBO (kader)
   - ○ MULO / MAVO / MBO (theoretisch)
   - ○ HAVO
   - ○ VWO
   - ○ Bachelor HBO/WO
   - ○ Master
   - ○ Doctoraat

Roken

3. Rookt u, of heeft u gerookt?
   - ○ Nee
   - ○ Ja, ik rook vanaf mijn ___ e jaar
   - ○ Gestopt. Ik heb gerookt van mijn___ e jaar tot mijn ___ e jaar

   Zo ja, ik rook/rookte
   - ○ sigaretten, ongeveer ___ per dag
   - ○ sigaren, ongeveer ___ per dag
   - ○ pijp, ongeveer ___ keer per dag

Alcohol

4. Drinkt u alcohol?
   - ○ Nee
   - ○ Ja, ongeveer ___ glazen per week
   - ○ Gestopt. Ik dronk van mijn ___ e jaar tot mijn ___ e jaar.
     Dit was ongeveer ___ glazen per dag.

Voedingssupplementen

5. Neemt u voedingssupplementen/ vitaminepreparaten?
   (bijvoorbeeld vitamine bruistabletten of magnesium)
   - ○ Nee
   - ○ Ja namelijk: __________________ (merk) ____ keer per week
     __________________ (merk) ____ keer per week
     __________________ (merk) ____ keer per week
     __________________ (merk) ____ keer per week

Eetgedrag

6. Hoe vaak eet u donkergroene bladgroente (b.v. spinazie, kool)?
   (Hier telt ook tussendoor mee of bijvoorbeeld op brood)
   - ○ Elke dag
   - ○ 4 – 6 keer per week
   - ○ 3 of minder keer per week

   De portie groente bedraagt dan ongeveer:
   - ○ 1 opscheplepel
   - ○ 2 opscheplepels
   - ○ 3 opscheplepels
   - ○ 4 opscheplepels

7. Hoe vaak eet u rode/gele groente (b.v. paprika, wortel)?
   (Hier telt ook tussendoor mee of bijvoorbeeld op brood)
   - ○ Elke dag
   - ○ 4 – 6 keer per week
   - ○ 3 of minder keer per week

   De portie groente bedraagt dan ongeveer:
   - ○ 1 opscheplepel
   - ○ 2 opscheplepels
   - ○ 3 opscheplepels
   - ○ 4 opscheplepels

8. Hoe veel fruit eet u ongeveer per week?
(fruit in sap, telt hier niet bij mee)
- ○ Elke dag meerdere porties
- ○ Elke dag 1 portie
- ○ 4 – 6 porties per week
- ○ 3 of minder porties per week

9. Hoe vaak eet u vis?
- ○ Meermaals per week
- ○ 1 keer per week
- ○ Af en toe (1 – 3 keer per maand)
- ○ (Bijna) nooit

10. Volgt u een bepaald dieet?
- ○ Nee
- ○ Vegetarisch
- ○ Veganistisch
- ○ Anders namelijk: ______________________

Lichaamsbeweging

11. Hoe zit het met uw lichaamsbeweging en lichamelijke activiteit?
- ○ Ik kan amper zelfstandig bewegen
- ○ Ik beweeg wel wat maar niet al te veel
- ○ Ik kan wandelen of fietsen in een normaal tempo
- ○ Ik kan stevig doorwandelen of fietsen
- ○ Ik sport intensief

Erfelijke aandoeningen

12. Het kan zijn dat u bij de oogarts gekomen bent vanwege glaucoom. Zijn er in uw familie (meer) personen met glaucoom?
(zo ja, vul dan in bijvoorbeeld vader, zus, neef)
- ○ Nee
- ○ Ja, namelijk: ______________________________

____

____

13. Zijn er in uw familie personen met andere oogheelkundige aandoeningen
(zo ja, vul dan in bijvoorbeeld vader, zus, neef)
- ○ Nee
- ○ Ja, namelijk: (familielid)______ (ziekte)________

________________ ___________________

________________ ___________________

________________ ___________________

________________ ___________________

________________ ___________________

Overige aandoeningen

14. Wordt u behandeld of bent u behandeld geweest voor:
- ○ Diabetes (Suikerziekte)
- ○ Kanker
- ○ Longziekten
- ○ Hartaanval / vaatziekten
- ○ Hersenbloeding
- ○ Hoge bloeddruk
- ○ Reuma
- ○ Anders, namelijk: ______________________

Medicatie

15. Gebruikt u medicatie van bv de drogist, dus niet op arts recept (bijvoorbeeld homeopathische middelen, paracetamol, aspirine)?
- ○ Nee
- ○ Ja namelijk: __________________ (merk) ____ keer per week

__________________ (merk) ____ keer per week

__________________ (merk) ____ keer per week

__________________ (merk) ____ keer per week

16. Geeft u toestemming om uw medicatielijst bij de apotheek op te vragen?
- ○ Nee
- ○ Ja

Hartelijk dank voor het beantwoorden van deze vragenlijst.
Indien u nog overige op/aanmerkingen heeft betreffende uw gegevens of niet genoeg mogelijkheden had bepaalde vragen te beantwoorden kunt u dit hier nog vermelden:
