# Supplementary material for: Plasma GDF-15 concentration is not elevated in open-angle glaucoma
Source: PLoS One. 2021 May 28;16(5):e0252630. doi: 10.1371/journal.pone.0252630 (PMC8162581; doi:10.1371/journal.pone.0252630)
Supplement: S2 File — (DOCX) [file pone.0252630.s006.docx]

**S references**

**1**. Kucharská J. Vitamins in Mitochondrial Function. In: Gvozdjáková A, editor. Mitochondrial Medicine: Mitochondrial Metabolism, Diseases, Diagnosis and Therapy. Dordrecht: Springer Netherlands; 2008. p. 367-84.

**2**. Eckert A. Mitochondrial effects of Ginkgo biloba extract. Int Psychogeriatr. 2012;24 Suppl 1:S18-20

**3**. Tian G, Sawashita J, Kubo H, Nishio SY, Hashimoto S, Suzuki N, et al. Ubiquinol-10 supplementation activates mitochondria functions to decelerate senescence in senescence-accelerated mice. Antioxid Redox Signal. 2014;20(16):2606-20

**4**. Ames BN, Atamna H, Killilea DW. Mineral and vitamin deficiencies can accelerate the mitochondrial decay of aging. Mol Aspects Med. 2005;26(4-5):363-78

**5**. Li L, Yang X. The Essential Element Manganese, Oxidative Stress, and Metabolic Diseases: Links and Interactions. Oxid Med Cell Longev. 2018;2018:7580707
